# Development and Evaluation of qPCR Detection Method and Zn-MgO/Alginate Active Packaging for Controlling *Listeria monocytogenes* Contamination in Cold-Smoked Salmon

**DOI:** 10.3390/foods9101353

**Published:** 2020-09-24

**Authors:** Priya Vizzini, Elena Beltrame, Valentina Zanet, Jasmina Vidic, Marisa Manzano

**Affiliations:** 1 Department of Agriculture Food Environmental and Animal Sciences, University of Udine, 33100 Udine, Italy; vizzini.priya@spes.uniud.it (P.V.); beltrame.elena@spes.uniud.it (E.B.); zanet.valentina@spes.uniud.it (V.Z.); 2 Micalis Institute, INRAE, AgroParisTech, Université Paris-Saclay, 78350 Jouy-en-Josas, France

**Keywords:** *Listeria monocytogenes*, cold-smoked salmon, Zn-MgO nanoparticles, active packaging, qPCR

## Abstract

To answer to food industry requests to monitor the presence of *L. monocytogenes* in cold-smoked salmon samples and to extend their shelf-life, a qPCR protocol for the detection of *L. monocytogenes*, and an antibacterial active packaging reinforced with zinc magnesium oxide nanoparticles (Zn-MgO NPs) were developed. The qPCR allowed the sensitive and easy detection of *L. monocytogenes* in naturally contaminated samples, with specificity in full agreement with the standard methods. The halo diffusion study indicated a high antibacterial efficiency of 1 mg/mL Zn-MgO NPs against *L. monocytogenes*, while the flow cytometry showed only moderate cytotoxicity of the nanoparticles towards mammalian cells at a concentration above 1 mg/mL. Thus, the novel active packaging was developed by using 1 mg/mL of Zn-MgO NPs to reinforce the alginate film. Cold-smoked salmon samples inoculated with *L. monocytogenes* and air-packed with the Zn-MgO NPs-alginate nanobiocomposite film showed no bacterial proliferation at 4 °C during 4 days. In the same condition, *L. monocytogenes* growth in control contaminated samples packed with alginate film alone. Our results suggest that Zn-MgO nanoparticles can extend the shelf-life of cold-smoked salmon samples.

## 1. Introduction

Foodborne diseases are responsible worldwide for millions of illnesses and thousands of hospitalizations that can be fatal, which demonstrates the urge to develop novel technologies to improve food safety. As such, rapid and accurate pathogen detection in foodstuffs together with advanced antibacterial food packaging play an important role in providing safe and fresh-like foods with longer shelf life. This is particularly true for ready-to-eat (RTE) foods that are associated with serious microbial risks because they are more susceptible to contamination by spoilage microorganisms and pathogens such as *Listeria monocytogenes*. Notably, RTE meat and fish products that undergo long-term refrigerated storage are considered as high-risk for *L. monocytogenes*.

*L. monocytogenes* is a short, motile, Gram-positive, non-spore-forming rod, invasive pathogen responsible for listeriosis, a foodborne disease associated with 30% cases of deaths [1,2,3]. *L. monocytogenes* possesses great resistance to low temperature, acidification, and to various antimicrobial agents used in food processing or equipment cleaning. For instance, bacteria can multiply and grow under salt concentrations up to 10% and at refrigeration temperatures (in the range of 4 °C to 8 °C) which makes it capable to overcome the various procedures set to limit microbial growth [4,5]. In addition, *L. monocytogenes* is one of the major causes of food recalls, causing large economic losses [1,6,7,8,9].

The difficulty to detect this bacterium during food-processing has led the food industry to search for novel, easy and accurate methods that would enable routine microbiological screening for *L. monocytogenes* food contamination [10,11]. Traditional plate count methods, such as the ISO standard (11290: 2017) method or its equivalents, require a long time for the analysis [12,13]. These methods consist of two enrichment and selective steps followed by bacterial isolation on differential media and biochemical tests for identification [14]. Thus, culture-independent methods for the rapid detection of *Listeria*, such as PCR-based methods (end-point PCR, PCR-RE, qPCR), are needed to reduce the analysis time and improve routine food control [10,14,15,16,17,18,19].

An inhibition or retardation of *L. monocytogenes* growth in RTE foods is also necessary because this pathogen can contaminate food products after the preparation step (during processing, storing and distribution) and continue to grow during shelf life which potentially leads to food spoilage and risk of foodborne diseases. One innovative way to inhibit *L. monocytogenes* growth in food during long-term storage is the use of active packaging (1935/2004/EC, 450/2009/EC). An active antimicrobial packaging targets specific microorganisms and controls the microbial population by releasing biocide substances onto or around the food, which assures food safety, extends shelf life and delays or prevents spoilage [20]. Metal oxide nanoparticles (NPs) are attractive biocide materials for active packaging because they are stable and highly efficient against various pathogenic bacteria [21,22]. Among the metal oxide NPs, zinc oxide (ZnO) NPs are being used industrially for many applications due to its strong antimicrobial effect on a broad spectrum of microorganisms and its easy incorporation into different matrices [23]. Moreover, ZnO is currently listed by FDA (U.S. Food and Drug Administration) as a generally recognized as safe material [24]. However, in vitro and in vivo studies in animal and human models indicated that nanosized ZnO could also be potentially hazardous due to its high reactivity [25,26]. In contrast, mixed Zn-MgO nanomaterials, which are produced through the association of zinc with highly biocompatible magnesium oxide (MgO) NPs, retained a strong antibacterial effect toward Gram-positive bacteria similar to ZnO NPs and was safe for mammalian cells like the nano-MgO [26,27,28]. In consequence, incorporation of mixed Zn-MgO NPs into an active packaging instead of pure nano-ZnO seems to be more suitable.

The aim of this work was to develop a fast, specific and easy qPCR protocol to test for the presence of *L. monocytogenes* in sliced cold-smoked salmon (CSS) samples, and to evaluate the utilization of Zn-MgO NPs to create an active film packaging in order to prolong CSS shelf life. CSS was chosen as a model food because it is distributed as an RTE food that does not undergo heat treatments before being consumed and is therefore considered of high risk of *L. monocytogenes* contamination [6,7,8,9].

## 2. Materials and Methods

### 2.1. Materials and Reagents

All media and reagents for microbiological analyses were purchased from Oxoid (Milan, Italy). Triton X-100, SDS, NaCl, Tris-HCl, sodium alginate, and EDTA were purchased from Sigma-Aldrich (Milan, Italy).

The AmpliTaq^®^ Buffer 10X, AmpliTaq^®^ DNA Polymerase, PCR Nucleotide Mix 10 mM and MgCl_2_ 25 mM were purchased from Applied Biosystems (Milan, Italy). The SsoFast™ EvaGreen^®^ kit (Bio-rad, Irvine, CA, USA) and MgCl_2_ 25 mM (Applied Biosystems, Milan, Italy) were employed to perform qPCR tests using the Rotor-Gene Q (Qiagen, Milan, Italy). Zn-MgO nanoparticles (Mg_1−*x*_Zn*_x_*O, x = 0.85), with an average size from 5 to 10 nm, used in this work were a kind gift from Slavica Stankic (INSP, France). They were prepared and characterized as described previously [28,29].

The ready-to-eat (RTE) food corresponds to 16 samples of 100 g of sliced cold-smoked salmon (*Salmo salar*), (CSS) that were purchased from local supermarkets and were processed from fresh never frozen fish. All the CSS samples had Aw values between 0.983 and 0.964, a pH about 6, and were preservative-free and vacuum-packaged. The reference bacteria used in this work are listed in Table 1.

### 2.2. Primer Design

A specific reverse primer (MarB) was designed using the “Multiple sequence alignment with hierarchical clustering” [30] and coupled to the Mar1 fw primer [15] for the specific detection of *L. monocytogenes* in food samples using qPCR technique. The couple of primers that were expected to produce an amplicon of 160 bp were tested in silico by confirming the production of the expected 160 bp amplicon with OligoAnalyzer 3.1 [31] and AmplifX 1.7.0 [32], before being used on DNAs extracted from the bacteria listed in Table 1.

### 2.3. PCR and qPCR Analyses

DNAs were extracted from the bacteria listed in Table 1 using the protocol described by Comi et al. [33] with some modifications. Briefly, 2 mL of homogenates were collected from the Stomacher bags with Bolton broth, centrifuged for 10 min at 13,000 rpm, and the pellet suspended in 200 µL breaking buffer (2% Triton X-100, 1% SDS, 100 mM NaCl, 10 mM Tris-HCl, 1 mM EDTA pH 8; Sigma-Aldrich) before being subjected to DNA extraction.

DNAs were used in an end-point PCR to verify the specificity and sensitivity of the primers and in qPCR to build the standard curve. The PCR protocol adopted was as follows: 5 μL AmpliTaq^®^ Buffer 10X (Applied Biosystems, Milan, Italy), 1 μL MgCl_2_ 25 mM (Applied Biosystems), 1 μL PCR Nucleotide Mix 10 mM each (Applied Biosystems), 1 μL of each primer (Mar1 and MarB at 10 μM), 0.25 μL AmpliTaq^®^ DNA Polymerase 5 units/μL (Applied Biosystems) in a final reaction volume of 50 μL including 1 μL of template DNA at 100 ng/µL. A no-template control called NTC was used to ensure the absence of contamination. Thermal cycler conditions consisted of 95 °C denaturation for 5 min, 35 cycles of 95 °C for 1 min, 48 °C for 45 s, 72 °C for 30 s and a final extension at 72 °C for 7 min in a Thermal Cycler (C1000 Touch™; Bio-rad, Hercules, CA, USA).

A qPCR using Mar1 and MarB primers and decimal dilutions (from 100 ng/µL to 1 fg/µL) of the DNA of *L. monocytogenes* 1/2b DISTAM was used to build a standard curve by relating fluorescence with threshold cycle (Ct). The threshold limit setting was performed in automatic mode using SsoFast^™^ EvaGreen^®^ kit (Bio-rad, Milan, Italy) according to the manufacturer’s instructions. The reaction mixture contained the following reagents: 10 μL SsoFast™ EvaGreen^®^ Supermix 2X (Bio-rad), 1 μL MgCl_2_ 25 mM (Applied Biosystems, Monza, Italy), 1 μL of each primer at 10 μM in a final reaction volume of 20 μL (13 μL of reaction mixture, 6 μL of nuclease-free water and 1 μL of template DNA at various concentrations). For each assay, a positive control using *L. monocytogenes* 1/2b DISTAM, negative control using *L. innocua* DSMZ 20649, and an NTC (1 μL water instead of DNA) were included. The thermal program applied consisted of 98 °C hot-start activation for 2 min, 40 cycles of 98 °C denaturation for 5 s and 60 °C annealing/extension for 20 s, followed by melting temperature analysis performed by gradually increasing the temperature from 60 to 95 °C (+0.5 °C/5 s) in the Rotor-Gene Q (Qiagen, Milan, Italy).

### 2.4. Plate Count Analyses of Cold-Smoked Salmon Samples

Sixteen samples (10 g each) of sliced salmon were added to a sterile Stomacher bag containing 40 mL of saline-peptone water (8 g/L NaCl, 1 g/L bacteriological peptone; Oxoid), homogenate in a Stomacher (PBI, Milan, Italy) for 30 s and used for plate count bacterial enumeration. Eight samples were analyzed immediately after purchasing (CSS 1-8 *t*_0_) and eight others were incubated at +4 °C and analyzed after 28 days (close to expiration date) (CSS 1-8 *t*_28_). The plate count method was used for the determination of: (i) total viable count on TSA, (Tryptone Soya Agar, Oxoid, Milan, Italy) incubated at 30 °C for 48 h; (ii) Enterobacteriaceae, count on VRBG agar (Oxoid) incubated at 37 °C for 24 h; (iii) lactic acid bacteria (LAB) count on MRS (Oxoid) incubated at 30 °C for 48 h in Anaero-Jar 2.5 L (Oxoid) with microaerophilic conditions; (iv) yeasts and molds were enumerated on Malt Extract Agar (Oxoid) with 10 µg/mL of tetracycline (Sigma-Aldrich) incubated at 30 °C for 48 h.

For this, 10 g of the samples were transferred into a filter sterile Stomacher bag containing 40 mL, and were mixed in a Stomacher (PBI, Milan, Italy) for 30 s. The homogenization solutions were used for plate count bacterial enumeration and for DNA extraction. The *Listeria* Precis™ method (NF Validation, 2013, [34]) and molecular methods were used to detect the presence of *Listeria monocytogenes*. Twenty-five g of CSS samples were transferred into a filter sterile Stomacher bag, added with 225 mL of ONE Broth-*Listeria* (Oxoid) mixed for 30 s in a Stomacher (PBI) and incubated at 30 °C for 24 h.

The selective isolation of *L. monocytogenes* was performed according to *Listeria* Precis™ method. Briefly, 25 g of sample is enriched for 24 h ± 2 h at 30 °C in 225 mL ONE Broth-Listeria (Oxoid) and after 10 μL are plated by streaking on Brilliance Listeria Agar. After 22–26 h at 37 °C incubation blue/green colonies with halos are confirmed using the O.B.I.S. mono test. Colonies which do not develop deep purple color are confirmed *L. monocytogenes*. Presumed colonies of *L. monocytogenes*, were tested with the O.B.I.S. mono test (Oxoid), and the confirmed colonies were subjected to DNA extraction (DNA- Isolates). DNA was also extracted from One Broth solutions immediately (DNA-OB 1-8 *t*_0_) and after 24 h (DNA-OB 1-8 *t*_24_) according to Cecchini et al. [35].

### 2.5. Artificially Contamination (AC) of Cold-Smoked Samples

Ten grams of CSS samples were exposed with *L. monocytogenes* 1/2b DISTAM, at concentrations of 10 cells/g (AC_10_), 10^2^ cells/g (AC_100_) and 10^3^ cells/g (AC_1000_) and kept at 30 °C for 30 min to allow the inoculum to soak into the food. After the contamination, samples were incubated at 30 °C for 24 h in 1:10 ONE Broth-*Listeria* (Oxoid), before being processed for DNA extraction (for qPCR analyses) and analyzed with the *Listeria* Precis™ method.

### 2.6. Listeria monocytogenes Inhibition Test Using Zn-MgO Nanoparticles

To verify the possible utilization of Zn-MgO NPs for the production of an active film intended to prolong the shelf life of the CSS samples, the antibacterial effect of the Zn-MgO NPs was tested on *L. monocytogenes* using the haloes test. An amount of 100 µL of the microbial suspension of *L. monocytogenes* grown in BHI broth until about 5 × 10^6^ cells/mL (a concentration corresponding to 0.5 McFarland) was spread onto the surface of BHI agar plates. Subsequently, 5 mm wells were created [36] into the agar and filled with 20 µL suspensions of Zn-MgO at 0.1 mg/mL, 0.5 mg/mL, and 1 mg/mL. Negative control of 20 µL water (0 mg/mL Zn-MgO) was used. The plates were incubated at 37 °C for 24 h, the diameters of the zones of inhibition (haloes) were measured using a ruler. Values were expressed in mm diameter, mean ± SD of at least three independent experiments.

### 2.7. Cytotoxicity Test on Mammalian Cell Cultures and FACS Measurements

Human macrophage-like cells U937 and differentiated human promyelocytic leukemia cell line HL-60 were used to estimate the cytotoxicity of Zn-MgO nanoparticles. Cells were cultured in RMPI 1640 medium (Lonza, Verviers, Belgium) supplemented with 10% fetal bovine serum, 2 mm l-glutamine, 100 IU/mL of penicillin, and 100 μg/mL of streptomycin [26,37], at 37 °C in a 5% CO_2_ incubator. After incubation with the NPs at 0 mg/mL, 0.1 mg/mL and 1 mg/mL, the cells were trypsinized to detach them from the culture vessel and concentrated by centrifugation (5 min at 3000× *g*). The collected cells were washed twice in PBS, resuspended in minimum culture medium containing acridine orange (0.1 μg/mL) and incubated in the dark, at 37 °C for 10 min. The stained cells were washed with PBS and fixed with 3.2% paraformaldehyde in PBS for 30 min. Flow cytometry analysis was performed using Becton FACSCalibur (Dickinson and Company, Franklin Lakes, NJ, USA) with the 488 nm laser line and FL-1 channel to quantify cell viability. Each analysis was done on 5 × 10^4^ cells in triplicate. Results are presented as mean ± SD.

### 2.8. Active Film Packaging Formation and Challenge Test Protocol

Active films with antibacterial activity prepared from Zn-MgO powder were produced following a modified procedure from Akbar and Kumar (2014) [38]. Briefly, 1.167 g of sodium alginate were gently mixed with 50 mL of deionized water using a stirrer at room temperature for 6 h. A volume of 1.5 mL calcium chloride (5% *w*/*v*) and 1.5 mL glycerol (100%) were added to the 50 mL alginate solution and mixed for 10 min. Then, 55 mg of Zn-MgO powder was added to the sodium alginate and mixed for 5 min. The obtained solution (1 mg/mL) was poured into a Petri dish (60 mm diameter; Steriplan^®^ Petri dish, DURAN^®^) and evenly spread with the help of a slight rotation of the plate. Plates were incubated at 50 °C for 12 h, subsequently wet with 10 mL calcium chloride (5%) for 10 min and washed three times with distilled water before being dried at 50 °C for 1 h. A film without the addition of Zn-MgO was used as a negative control. Films were sterilized under ultraviolet radiation for 2 h to eliminate any surface contamination before their utilization.

Ten pieces of 1.5 cm × 1.5 cm (2.25 cm^2^) of CSS samples were subjected to a UV light treatment of 2 h (for surface sterilization) before utilization. Two pieces were immediately analyzed for the presence of *L. monocytogenes*, while eight pieces were inoculated with 100 μL of *L. monocytogenes* overnight culture diluted at 1.3 × 10^9^ CFU/mL to obtain an inoculum of 6.0 × 10^2^ CFU/cm^2^. Four inoculated samples were packed with the active alginate film (with the addition of NPs) and four with the standard alginate film (no addition of NPs). All inoculated samples were stored at +4 °C for 4 days (typical smoked salmon storage temperature) before being analyzed. Packaging and storing were performed under aerobic conditions. Data are expressed as (mean ± SD) CFU/cm^2^.

## 3. Results and Discussion

### 3.1. Plate Count Results on Commercial CSS RTE CCS and Artificially Contaminated CSS Samples

As expected, the AFNOR *Listeria* Precis™ method was able to detect *L. monocytogenes* in the artificially contaminated CSS samples (AC_10_, AC_100_, AC_1000_) at the three concentrations of 10, 100 and 1000 cells/g used in the assay. Thereafter, the microbiological load of the commercial RTE CSS immediately after purchasing and after 28 days of storage at 4 °C was obtained by using the plate count method immediately after purchasing and after 28 days of storage at 4 °C and is reported in Table 2. The microbiological data obtained were in agreement with the literature data for safe CSS [39,40]. After 28 days, only one out of the 16 cold-smoked salmon samples (CSS5*t*_28_) was positive to *L. monocytogenes* when analyzed with the AFNOR validated *Listeria* Precis™ method. This finding is in accordance with the low prevalence of the pathogen reported in the literature for a high sample size [6,7,41]. Moreover, all isolates on Brilliance *Listeria* agar were tested for *L. monocytogenes* with the O.B.I.S. method, before being tested by PCR.

### 3.2. Specificity and Sensitivity of the Primers With PCR and qPCR

**Figure 1:**
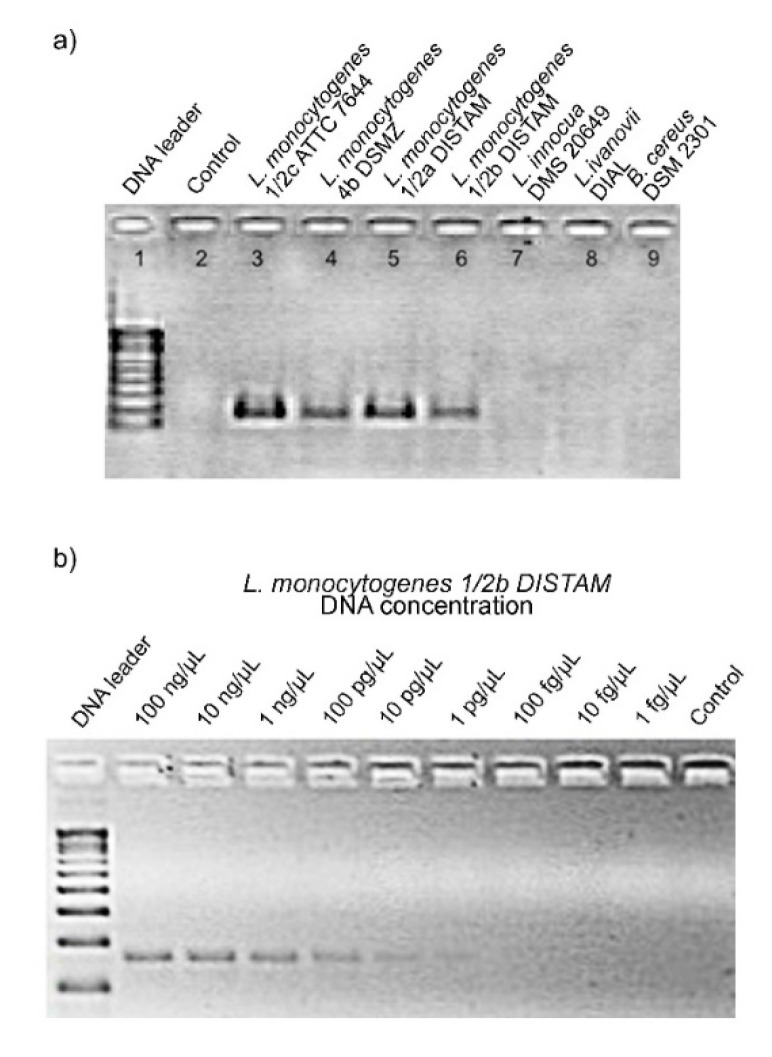
(**a**) PCR specificity with primers Mar1-MarB using positive and negatives controls. Line 1: 100 bp DNA ladder (Promega, Milan, Italy); line 2: NCT (no template control); line 3: *L. monocytogenes* 1/2c ATCC 7644; line 4: *L. monocytogenes* 4b DSMZ 15675; line 5: *L. monocytogenes* 1/2a DISTAM; line 6: *L. monocytogenes* 1/2b DISTAM; line 7: *L. innocua* DSM 20649; line 8: *L. ivanovii* DI4A; line 9: *Bacillus cereus* DSM 2301. (**b**) PCR sensitivity with Mar1-MarB using serial dilution of DNA of *L. monocytogenes* 1/2b DISTAM. Line 1: 100 bp DNA ladder (Promega, Italy); lines 2: 100 ng/μL DNA; line 3: 10 ng/μL DNA; line 4: 1 ng/μL DNA; line 5: 100 pg/μL DNA; line 6: 10 pg/μL DNA; line 7: 1 pg/μL DNA; line 8: 100 fg/μL DNA; line 9: 10 fg/μL DNA; line 10: 1 fg /μL DNA; line 11: NCT (no template control).

The PCR carried out on the DNA extracted from the reference strains, listed in Table 1, demonstrated the specificity of the primers Mar1-MarB. The expected amplicon of 160 bp was only produced by strains of *L. monocytogenes*, while no DNA bands were visible for other *Listeria* species or other bacterial genus tested (Figure 1a). The sensitivity of the primers Mar1-MarB with the endpoint method used was of 1 pg/µL DNA of *L. monocytogenes* as shown in Figure 1b. The good result of Mar1-MarB primers on specificity obtained with endpoint PCR justified their use in a quantitative PCR.

The standard curve of the qPCR obtained with primers Mar1-MarB showed a *R*^2^ = 0.997, a slope of −2.956, and an efficiency = −1 + 10(−1/slope) (Y = −2975 × log(conc) + 22.278). The efficiency value of 117% was calculated based on the obtained threshold cycles (Ct). In qPCR analysis, only efficiencies ranging from 90% to 110% are considered optimal. However, we can consider our efficiency value as good because it is similar to another efficiency that has been also reported as good during the quantification of various pathogens [42,43].

No positive samples for *L. monocytogenes* were detected using DNA extracted at *t*_0_ (DNA-OB *t*_0_ samples) in 16 CSS samples with qPCR absolute quantitation using the DNA extracted after enrichment in ONE Broth-*Listeria*. Analysis of DNA extracted at *t*_28_ (DNA-OB*t*_28_ samples) indicated that only the sample CSS5*t*_28_ was positive to *L. monocytogenes* (Ct 25.61). The obtained results were in full agreement with those reported using AFNOR validated Listeria Precis™ method presented above. *L. monocytogenes* was quantified in artificially contaminated (AC) samples, after 24 h of enrichment step in ONE Broth-Listeria, to confirm the capability of the protocol to detect low bacterial amounts. The C_t_ value of AC_10_, AC_100_ and AC_1000_ was 23.12, 21.50 and 19.85, respectively. The correlation between C_t_ values and bacterial concentration in AC samples enabled the estimation of *L. monocytogenes* in the naturally contaminated sample CSS5*t*_28_ to concentration as low as 52.37 pg/µL, i.e., around 10 cells/g. This indicated the capability of the new qPCR procedure to detect a very low amount of the pathogen in a naturally contaminated sample.

### 3.3. Listeria monocytogenes Plate Inhibition Test

Before starting the elaboration of an active film containing Zn-MgO, the antibacterial effect of the nanoparticles toward *L. monocytogenes* was evaluated by the haloes test. The test was considered negative if the halo had the diameter of the well size (5 mm) and it was considered positive when its diameter was bigger than 5 mm. The negative control corresponding to the water solution containing no nanoparticle showed no halo. The absence of inhibition haloes was also observed for the solutions containing 0.1 and 0.5 mg/mL Zn-MgO. On the contrary, the solution of Zn-MgO at 1 mg/mL produced haloes of 8 ± 0.1 mm. This finding suggested the antimicrobial activity of Zn-MgO at 1 mg/mL against *L. monocytogenes*. Subsequently, the concentration of 1 mg/mL Zn-MgO was used to reinforce the alginate packaging films.

### 3.4. Zn-MgO Cytotoxicity

**Figure 2:**
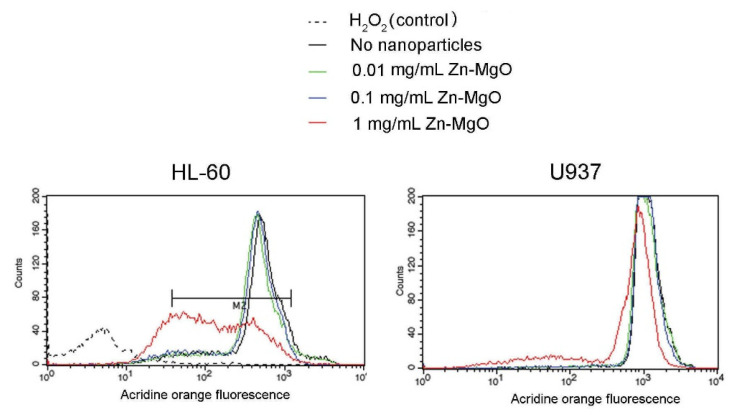
Viability of human monocyte U937 cells and human promyelocytic leukemia cells HL-60 incubated with various concentrations of Zn-MgO NPs for 24 h was estimated by acridine orange staining and flow cytometry analysis. H_2_O_2_ was used as a positive control as oxidative stress induces 100% mortality.

To verify whether Zn-MgO is toxic towards mammalian cells, U937, and HL-60 cells were incubated with the nanoparticles at different concentrations and analyzed by the acridine orange assay and flow cytometry (treated cells). In controls, cells were incubated with the medium without nanoparticles (untreated cells). After incubation, cells were stained by the acridine orange dye, which can enter mammalian cells and accumulate in lysosomes. Dead and damaged cells have ruptured lysosomes and are expected to show a decrease in the dye red fluorescence [26,27]. No reduction of acridine orange derived fluorescence intensity was observed in cells treated with 0.01 mg/mL or 0.1 mg/mL Zn-MgO NPs (Figure 2). Indeed, even if (3.1 ± 0.6)% of U937 dead cells was observed after treatment with 0.1 mg/mL Zn-MgO, this cell death was not significant as untreated U937 cells already presented (2.5 ± 0.8)% of cell mortality. HL-60 cells incubated with 0.1 mg/mL Zn-MgO shown about (17 ± 3.1)% of dead cells, while the mortality of untreated HL-60 was close to (14 ± 2.6)%, as shown in Figure 2. No significant increase in cell mortality was observed in U937 treated with 1 mg/mL Zn-MgO (about 11%), while HL-60 treated with 1 mg/mL Zn-MgO showed a partial cytotoxic effect because presented about (46 ± 6.3)% dead cells. In control experiments, cells treated with 3% H_2_O_2_ for 30 min were completely destroyed as shown for HL-60 cells (Figure 2). These results are in line with previous data published on Zn-MgO cytotoxic effect on HeLa cells and macrophages that were shown to tolerate metal oxide NPs to some extent (≤1 mg/mL) [26,27]. Probably mammalian cells are capable to phagocytose and/or digest metal oxide nanoparticles [25]. To conclude, our flow cytometry test, suggests that Zn-MgO at concentrations below 1 mg/mL may be safely used to form an active packaging.

### 3.5. Challenge Test with Zn-MgO Alginate-Nanobiocomposite Active Film

**Figure 3:**
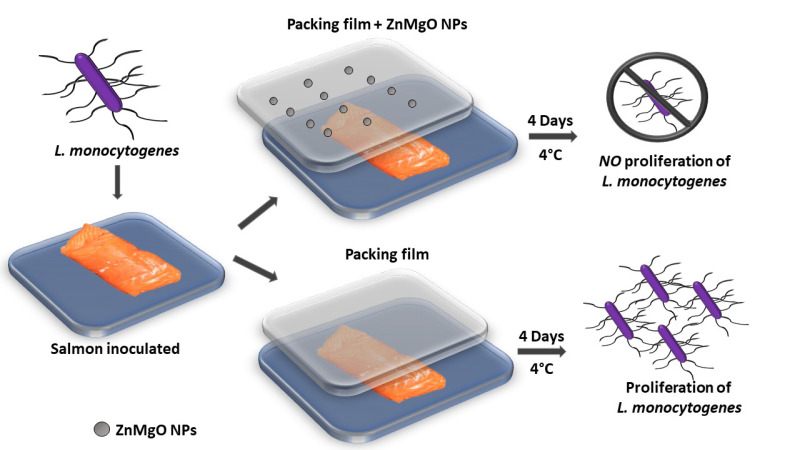
Description of the test conducted on cold-smoked salmon samples to verify active film effect vs. standard alginate film used for packing.

Figure 3 illustrates the working procedure in the challenge test with Zn-MgO used as an alginate active film reinforcement. The sodium alginate, used as a packaging material to prevent the detrimental effect of oxygen on food quality, is a biodegrading film-forming biopolymer, authorized as a food additive in the EC [44]. We chose to reinforce alginate with nano-Zn-MgO NPs, because of its ability to react with various divalent and trivalent cations.

CSS samples artificially contaminated with *L. monocytogenes* and packed with a pure alginate film, or with the film with incorporated Zn-MnO NPs were evaluated after storage at 4 °C for 4 days. In the control smoked salmon pieces that were not inoculated, *L. monocytogenes* was not detected. The inoculated samples packed with the Zn-MgO-nanobiocomposite active film showed a charge of (6.2 × 10^2^ ± 1.1 × 10^2^) CFU/cm^2^, while samples packed with the film without the addition of nanoparticles showed an increased *Listeria* charge value of about one log, (1.6 × 10^3^ ± 0.5 × 10^3^) CFU/cm^2^. These results suggest that 1 mg/mL nano-Zn-MgO provided an antibacterial activity to alginate film. Indeed, the lower charge of *L. monocytogenes* observed in the samples packed with the active film suggests that it is possible to extend the shelf life of the salmon.

The efficacy of metal oxide nanoparticles as bio-preservatives in polymer-based films is probably related to film direct contact with the food surface and in that way continuous release of the particles with antimicrobial activity onto the food surfaces [20,45,46]. The EFSA Panel on Food Contact Materials, Enzymes, Flavourings and Processing Aids (CEF Panel) evaluated the safety of nano-ZnO only based on the migration of soluble Zn^2+^-ions [7,47]. We can, thus, presume that the observed inhibition of *Listeria* proliferation in CSS samples packed with Zn-MgO-nanobiocomposite originated from the diffusion of Zn^2+^-ions from the film to salmon meat surface. However, the solubility and diffusion of metal ions in metal oxide nanopowders integrated into polymers and aqueous solutions were shown to be low [27,48,49,50]. Taking into account that no significant cytotoxic effect towards mammalian cells of Zn-MgO NPs at concentration <1 mg/mL was observed, the produced active packaging could be acceptable to consumers.

Previous studies have been reported on reinforcing natural polymers with antimicrobials (such as divergicin, nisin, potassium sorbate, sodium lactate) to produce active packaging films that suppress *L. monocytogenes* growth in CSS products [20,51]. It has been shown that the effectiveness of antimicrobial active films in various foods depends on the nature and concentration of the reinforcing agent used. Moreover, the polymer used may play a role. For instance, when enterocin was used to inhibit *L. monocytogenes* growth, its antimicrobial effects were found superior in alginate film than in zein and polyvinyl alcohol films [52]. Here, we showed that the addition of Zn-MgO nanoparticles to the alginate film could maintain the food product at a high level of quality for a longer period.

The EC Regulation 2073/2005 on microbiological criteria for RTE foods supporting the growth of *L. monocytogenes* recommends the concentration limit to 100 CFU/g at the end of the shelf life [52,53]. Zn-MgO NPs integrated into the biodegradable nanocomposite protecting film comply with this regulation and seem to be a good candidate for the preservation of RTE foods. However, the full validation of the Zn-MgO NPs-alginate active packaging will involve additional tests in order to characterize its effects on the proliferation of other pathogenic microorganisms and other RTE foods.

## 4. Conclusions

We showed that Zn-MgO NPs obtained by doping MgO crystal surface with Zn^2+^-ions possess improved biocompatibility together with high antibacterial activity. The packaging film obtained by the incorporation of Zn-MgO NPs in a biodegradable alginate nanocomposite prevented *L. monocytogenes* proliferation in refrigerated cold-smoked salmon in contrast to the control samples that were packaged with a pure alginate film. Additional studies with sensory and safety evaluation panels should be conducted before considering the commercialization of such films.

The qPCR method described here represents a contribution to the state of the art of the quantitative methods for *L. monocytogenes* detection in food samples. It offers the possibility to quantify as low as 10 CFU/g of the bacterium in CSSs after an enrichment step. The primers used in this work can help to reduce the time required to detect *L. monocytogenes* in RTE foods due to the high sensitivity in qPCR. The proposed protocol is useful for food safety at the industrial level because it can help to avoid food recalls by the rapid identification of the source of CSS contamination with *L. monocytogenes* and control of its correct elimination from the processing chain.

## Figures and Tables

**Table 1:** List of the microorganisms used.

	Microorganism	Reference Code
Positive controls	*Listeria monocytogenes* 1/2c	* ATCC 7644
	*Listeria monocytogenes* 1/2a	° DISTAM
	*Listeria monocytogenes* 1/2b	° DISTAM
	*Listeria monocytogenes* 4b	# DSMZ 15675
Negative controls	*Listeria innocua*	# DSMZ 20649
	*Listeria ivanovii*	§ DI4A
	*Salmonella enterica*	# DSMZ 9145
	*Enterobacter* spp.	§ DI4A
	*Escherichia coli*	§ DI4A
	*Bacillus cereus*	# DSMZ 2301
	*Campylobacter jejuni*	# DSMZ 49943
	*Lactobacillus plantarum*	* ATCC BAA-793
	*Lactobacillus rhamnosus*	* ATCC 53103
	*Lactobacillus paracasei*	# DSMZ 5622
	*Lactobacillus brevis*	# DSMZ 20054
	*Bacillus subtilis*	# DSMZ 4181

* ATCC: American Type Culture Collection (Manassas, VA, USA); ° DISTAM: Dipartimento di Scienze e Tecnologie Alimentari e Microbiologiche (Milan, Italy); # DSMZ: Deutsche Sammlung von Mikroorganism und Zellkulturen GmbH (Braunschweigh, Germany); § DI4A: Dipartimento di Scienze AgraroAlimentari, Ambientali e Animali dell’Università degli Studi di Udine (Udine, Italy).

**Table 2:** Plate count data of cold-smoked salmon (CSS) samples expressed as Colony Forming Unit (CFU)/g. *t*_0_: analysis at the purchasing; *t*_28_: analysis after 28 days incubation at +4 °C.

Samples	Total Viable	Lactic Acid	Enterobacteriaceae	Yeasts
	Count	Bacteria		
CSS1*t*_0_	6.25	4.94	<0.7	<1.40
CSS2*t*_0_	5.38	4.94	<4.34	4.59
CSS3*t*_0_	5.78	<4.34	1.65	2.89
CSS4*t*_0_	4.40	<4.34	<0.7	1.57
CSS5*t*_0_	6.08	4.80	3.65	3.25
CSS6*t*_0_	5.23	4.87	<0.7	1.40
CSS7*t*_0_	8.11	6.98	<0.7	4.77
CSS8*t*_0_	5.86	5	<0.7	4.50
CSS1*t*_28_	5.38	<4.40	<0.7	2.84
CSS2*t*_28_	7.84	7.18	4.30	5.99
CSS3*t*_28_	5.91	4.87	<0.7	3.84
CSS4*t*_28_	6.50	5.95	<0.7	5.75
CSS5*t*_28_	6.23	<4.38	1.72	2.41
CSS6*t*_28_	6.11	5.96	2.36	<1.40
CSS7*t*_28_	8.25	7.72	<0.7	5.18
CSS8*t*_28_	6.08	<4.34	<0.7	4.76
